# Early markers of airways inflammation and occupational asthma: Rationale, study design and follow-up rates among bakery, pastry and hairdressing apprentices

**DOI:** 10.1186/1471-2458-9-113

**Published:** 2009-04-23

**Authors:** Paul Tossa, Abraham Bohadana, Valérie Demange, Pascal Wild, Jean-Pierre Michaely, Bernard Hannhart, Christophe Paris, Denis Zmirou-Navier

**Affiliations:** 1 INSERM U954, School of Medicine, 9 avenue de la forêt de Haye, 54500 Vandoeuvre-Les-Nancy, France; 2 Nancy University Medical School, France; 3 INRS, Occupational Epidemiology Department, Paris, France; 4 Ecole des Hautes Etudes en Santé Publique (EHESP), Rennes, France

## Abstract

**Background:**

Occupational asthma is a common type of asthma caused by a specific agent in the workplace. The basic alteration of occupational asthma is airways inflammation. Although most patients with occupational asthma are mature adults, there is evidence that airways inflammation starts soon after inception of exposure, including during apprenticeship. Airways hyper responsiveness to methacholine is a valid surrogate marker of airways inflammation, which has proved useful in occupational epidemiology. But it is time-consuming, requires active subject's cooperation and is not readily feasible. Other non-invasive and potentially more useful tests include the forced oscillation technique, measurement of fraction exhaled nitric oxide, and eosinophils count in nasal lavage fluid.

**Methods and design:**

This study aims to investigate early development of airways inflammation and asthma-like symptoms in apprentice bakers, pastry-makers and hairdressers, three populations at risk of occupational asthma whose work-related exposures involve agents of different nature. The objectives are to (i) examine the performance of the non-invasive tests cited above in detecting early airways inflammation that might eventually develop into occupational asthma; and (ii) evaluate whether, and how, constitutional (e.g. atopy) and behavioural (e.g. smoking) risk factors for occupational asthma modulate the effects of allergenic and/or irritative substances involved in these occupations. This paper presents the study rationale and detailed protocol.

**Discussion:**

Among 441 volunteers included at the first visit, 354 attended the fourth one. Drop outs were investigated and showed unrelated to the study outcome. Sample size and follow-up participation rates suggest that the data collected in this study will allow it to meet its objectives.

## Background

Occupational asthma (OA) is a "*disease characterized by variable airflow limitation and/or airway hyper responsiveness due to causes and conditions attributable to a particular occupational environment and not to stimuli encountered outside the workplace*" [1,2]. Conventionally, two forms of OA have been described: the immunological type occurs after a latency period of exposure, necessary for the worker to acquire sensitization to the causal agent; this is the most common type of OA comprising about 90% of cases. An IgE-mediated mechanism has been described for exposure to high molecular weight (HMW) agents (>5 kDa; e.g. animal and flour proteins). In turn, the nature of the immunologic mechanism involved is not completely understood for exposure to low molecular weight (LMW) agents (<5 kDa; e.g. isocyanates or alkali persulfates) [3]. The second, non-immunologic form of OA is much less common, comprising only about 7% of cases [4]. It is characterized by the absence of a latency period and occurs typically after the accidental exposure to high concentrations of irritating vapor, gas or smoke. It corresponds to the "reactive airway dysfunction syndrome" (RADS) originally described by Brooks and colleagues [5]. About 300 agents, capable of causing OA, have been reported and registries of causal occupations and agents are widely available (e.g. ). In France, according to the national OA registry (ONAP), the most frequently incriminated agents are flour (20.3%), isocyanates (14.1%), latex (7.2%), aldehyde (5.9%), persulfate salts (5.8%), and wood dust (3.7%). The highest OA risks are encountered in bakers and pastry-makers (683 cases/million subjects), car painters (326/million), hairdressers (308/million), and woodworkers (218/million) [6]. From the pathogenic standpoint, the basic alteration of OA is airways inflammation [7]. In susceptible individuals, the inflammation causes recurrent episodes of wheezing, dyspnea, chest tightness and coughing, which are typically accompanied by widespread airflow obstruction and airways hyper responsiveness (AHR). Of practical importance is the fact that in most patients, symptoms and AHR persist even after removal from exposure, a finding ascribed to persistent airways inflammation and remodeling [8]. Although there is only limited information on the pre-morbid state – from beginning of exposure to onset of OA – it has been shown that the rate for acquiring sensitization and symptoms varies with the causative agent [9]. Furthermore, it is now becoming clear that the noxious effects of exposure might start soon, e.g. during the apprenticeship period, prior to employment. In a series of papers, Gautrin and colleagues [10-13] reported findings observed amongst a large cohort of apprentices (n = 769) starting careers in animal health, pastry-making and dental hygiene programs, who were examined at onset of exposure to HMW agents and monitored prospectively for up to 4 years. The authors observed a high incidence of work-related sensitization [11,14], probable occupational rhino-conjunctivitis [11] and OA [11,13], and a tendency for symptoms and diseases to occur mostly in the first 2–3 years after start of exposure [12]. In another study, the same team [10] found that immunological sensitization and asthma-like symptoms were the main determinants for leaving the training school. Generally, these data support the view, already suggested in earlier studies, that airways inflammation might be an early phenomenon in the chain of events leading to OA. Indeed, almost three decades ago, Thiel and Ulmer [15] found an increased prevalence of AHR in apprentice bakers compared with control subjects and concluded that AHR may precede a clinical outbreak of flour-induced OA. More recently, Kennedy and colleagues [16] supported this idea by demonstrating a significant association between exposure and increasing AHR in a group of 82 machine operator apprentices after a 2-year longitudinal follow-up period.

Several methods are now used to assess airways inflammation, but not all are equally suitable for studying populations. For example, bronchial biopsies from proximal airways – considered the "gold standard" – are invasive and impractical in population samples. In turn, AHR to non-specific stimuli, such as methacholine, is a valid surrogate marker of airways inflammation [17] and has proved useful in assessing the effects of occupational exposure [18,19]; however, this test is time-consuming, necessitates subject's cooperation and is not readily available. Alternatively, non-invasive strategies for assessing airways function and inflammation have given renewed relevance to three techniques: the forced oscillation technique (FOT) [20], measurement of fraction of exhaled nitric oxide [21] (FE_NO_) and eosinophils count in nasal lavage fluid [22]. FOT permits measurement of airways resistance and its usage implements the same rationale as spirometry. FE_NO _is an inflammation marker, which is increased in asthma patients [23,24], but is curtailed by corticosteroid treatment and has been found to correlate with both eosinophilic airways inflammation and AHR [25]. Finally, use of eosinophils counting in nasal lavage fluid is based on the "one airway, one disease" concept [26] according to which asthma and rhinitis – which often coexist in occupational exposure – are considered part of a continuum of inflammation within one common airway. These methods have been validated, are well tolerated and are reproducible [22,27,28], which make them good candidates for use in occupational epidemiology. Moreover, they are not time-consuming and are less dependent on subject cooperation. Surprisingly, only few epidemiological studies [29] have reported such methods of OA investigation.

Mindful of the above considerations, we decided to investigate the early development of airways inflammation and asthma-like symptoms in a cohort of apprentice bakers and hairdressers, two populations at high risk of OA. Our aim was twofold: a) to examine the performance of a battery of non invasive tests likely to detect early airways inflammation that might eventually develop into OA; and b) to better understand the influence of both constitutional (e.g. atopy) and behavioral (e.g. smoking) risk factors on the "natural history" of airways inflammation and OA. In this report we present the rationale and detailed protocol of this study, and demonstrate its feasibility.

## Study protocol

### Subjects and study design

This study comprises a prospective follow-up of apprentices over their 2-year apprenticeship period. All apprentice bakers, pastry-makers and hairdressers starting career programs at six vocational schools in Lorraine, North-Eastern France, were invited to take part in the study. Those who accepted were included, provided they had a history neither of previous exposure to substances known to induce OA, nor of asthma diagnosed by a physician. Examinations were carried out at the Clinical Investigations Center (CIC) of the French national health institute at Nancy University Hospital. The research program was authorized by the local ethics committee and written consent was obtained from either the apprentices themselves (18 years or older) or their parents (< 18 years). Following a first study inclusion examination which took place about three months after the beginning of the training programme, three follow-up examinations were scheduled 3, 9 and 12 months after examination 1, respectively.

The desired sample size of approximately 400 to 450 subjects was designed to ensure 70 to 80% sensitivity to the new inflammation tests with 10–15% accuracy (α = 5%), based on an expected 10–15% airways inflammation incidence and a 25% drop-out of students during the 2-year follow-up period.

### Recruitment procedure

Apprentices were initially informed of the study at their vocational training centers, soon after entry, and were invited to participate. Most were minors at the start of the study. A letter with an enclosed consent form was sent to parents to request their approval. Only subjects whose signed consents had been returned were asked to undergo medical examinations. Parents or students were called by telephone, if they had not returned their consent forms three weeks after mailing. Apprenticeship schools cooperated and groups of volunteer students were then transported from their school to the CIC by minivan or taxi. Examinations lasted approximately 2 hours to be all completed for each student, in a standardized order. Five to 10 volunteers were examined each day. Because training schools could be up to 100 km away from where examinations were conducted, students were absent from school during a whole day (8:30 AM to 4:00 PM, on average). They were offered lunch. According to when the appointment could be set for a given student during the training programme at school, the visits took place between 3 days to 3 weeks after last exposure at the bakery, pastry or hairdressing saloon where he/she practiced.

### Clinical examination and questionnaire

On arrival, subjects filled in a questionnaire concerning respiratory symptoms and a detailed active and passive smoking history [30]. In order to study their incidence along the training programme, symptoms were assessed at each visit using a standardized questionnaire covering personal and demographic information, past chest diseases and symptoms and past and present smoking habits. Work-related symptoms were considered present if the subject answered positively the question(s) « Have you ever had complaints at work of: (a) irritant, dry cough; (b) runny nose, stuffy nose, sneezing; (c) breathlessness; (d) chest wheezing ». A "yes" answer should be followed by a positive answer to the question « Do these complaints disappear, when you leave work (evening, weekends, or holidays)? ». Rhinitis-like symptoms were defined as the presence of at least one of the nasal symptoms cited above with or without eye's symptoms. An asthma-like symptom was defined as at least two positive answers to questions on wheezing, chest tightness, or shortness of breath under usual conditions or under conditions such as exercise, exposure to cold air, strong smells, smoke and dust. Because of the influence of cigarette smoking on exhaled nitric oxide level, smoking habits were recorded at each visit. *Non-smokers *were defined as subjects, who had never regularly smoked one or more cigarettes a day or had smoked one or more cigarettes a day for less than one year. *Current smokers *were defined as subjects, who reported smoking regularly one or more cigarettes a day for at least one year. *Past-smokers *were subjects, who reported smoking regularly one or more cigarettes in the past, but who had given up smoking prior to the study [19] Finally, a physical examination was performed, during which special attention was given to ocular, upper and lower respiratory tract and cutaneous signs of allergic diseases. Furthermore, lung sounds were carefully assessed by auscultation and the presence of adventitious sounds – especially wheezing sounds – was recorded.

### Fraction exhaled nitric oxide (FE_NO_)

Immediately after the clinical examination, FE_NO _was measured in compliance with ATS/ERS recommendations [31] and expressed in parts per billion (ppb). Measurements were taken by a trained technician using a chemiluminescence analyzer (NIOX^® ^2.0 system; Aerocrine AB, Solna, Sweden). The subject was tested in a sitting position and exhaled against an oral pressure of 5 cm H_2_0 at a flow rate of 50 mL/s with a sustained 10s plateau. An oral pressure of 5 cmH_2_0, enough to close the velum was applied to prevent nasal NO contamination. Three correctly performed exhalations were recorded during each session. Any exhalation not meeting ATS/ERS requirements was rejected by the NIOX system.

### Pulmonary function tests

Pulmonary functions tests were conducted after FE_NO _measurement, using a Random-noise Oscillatory Spirometer (R.O.S) system combining respiratory impedance by Forced Oscillation Technique (FOT) and spirometric forced expiration measurements in the same unit (SensorMedics Corporation, Datalink, Montpellier, France). Total impedance of the respiratory system (Z_rs_) by FOT was systematically measured prior to spirometry to avoid undesirable effects of forced expiratory maneuvers (e.g. cough, bronchoconstriction). Briefly, pseudo random pressure variations from 4–30 Hz generated by a loudspeaker were applied at the mouth, superimposed on spontaneous breathing. Mouth pressure was recorded by a differential pressure transducer (Honeywell 176 PC ± 35 hPa pressure transducer (Microswitch, Boston, MA, USA) and airway flow by a Fleisch No. 2 pneumotachograph [Metabo, Epalinges, Switzerland]) connected to a similar transducer with a matched frequency response. Signals were digitized by a computer at a frequency of 128 Hz for periods of 16 s and their fast Fourier transform (FFT) was computed by blocks of 256 points with 50% overlap. Impedance data from three reproducible measurements without obvious artifacts were averaged. Z_rs _was partitioned into a real, resistance-related part (R_rs_) and an imaginary reactance-related part (X_rs_). R_rs _was computed as the ratio of pressure and flow phase components and reflects the respiratory system (airways, lung tissue and chest wall) resistance properties. In turn, X_rs _is influenced by the elasticity and mass inertia of airways, lung tissue, thorax and inertia of the air within the bronchi To avoid artifactual errors, only impedance values with a coherence function γ^2 ^equal to or exceeding 0.95 were retained. This function was decreased in the presence of noise or nonlinearity in the relation of the pressure and flow signals.

***Spirometry ***was undertaken in the sitting position with the system operating in spirometry mode. Forced vital capacity (FVC), forced expiratory volume in one second (FEV_1_) and maximal expiratory flows at various lung volumes (V'max) were obtained by having the subject expire forcefully after a maximum inspiratory maneuver. At least three forced expiratory maneuvers, satisfying the recommended criteria (ATS 1995) [32] were recorded as baseline measurement. The largest FVC and FEV_1 _values were retained for analysis. Results were expressed as a percentage of the predicted values given by the European Steel and Coal Community Working Party [33].

### Airways responsiveness to methacholine

Non specific airways responsiveness was evaluated using the methacholine challenge test (MCT) based on a previously described protocol [18,19]. The highest FEV_1 _from at least three acceptable maneuvers was used as baseline measurement. With a nose clip in place, the subject was asked to inhale three cumulative doses of methacholine (100, 600 and 1600 μg i.e. 0.5, 3.0 and 8.0 μmol respectively) administered in succession, using an ATOMISOR AD dosimeter equipped with a NL11D nebulizer delivering fixed doses of 50, 100 or 200 μg (i.e. 0.25, 0.50 or 1.0 μmol) respectively of methacholine per breath. The sequence – methacholine inhalation, impedance measurement and spirometry – was repeated until the last dose of methacholine was inhaled or when FEV_1 _fell by 20% or more below the baseline value. Subjects who experienced a fall in FEV_1 _of > 20% were classified as having a positive MCT (MCT+). For these subjects, the challenge was terminated by inhalation of 200 μg of salbutamol. It was expected that most subjects would fail to experience this specific response, so an additional, non-censored responsiveness index was calculated. This was the linear two-point dose-response slope (DRS) for FEV_1_, calculated as a percentage decrease in FEV_1 _after the last dose divided by the total dose of methacholine administered [34]. To avoid zero or negative values, a constant of +2.5 was added to all DRS values and the values were normalized and thereafter expressed as NDRS [19]. Occurrence or aggravation of AHR, taken altogether as the main outcome, were defined as:

- a 20% or more decrease of FEV1 at any visit during the MCT when the test was negative at inclusion (MCT-), even if subjects experienced MCT- at a further visit; or

- a 20% or more decrease of FEV1 at at the first, followed at a subsequent visit by the same decrease in FEV1 but at a lower dose of methacholine (aggravation); or

- a decrease by 0.100 or more of the NDRS at any visit compared to the NDRS measured at the first visit, if at this visit FEV1 decreased by a minimum of 15%. The 0.100 cut-off point was the mean decrease in all MCT+ subjects (aggravation).

### Eosinophil count in nasal lavage fluid

Nasal lavage was performed using Hilding's method [22]. Briefly, 5 ml of normal saline solution (9‰, 37°C) were instilled in the nostril through a Foley catheter with an inflated balloon (5–8 mL of air) to ensure air tightness. The saline solution was kept in contact with the nasal mucosa and three cycles of instillation/aspiration were executed with the same solution. The procedure was repeated on the opposite nostril; the two lavage fluids were collected in the same tube. The sample was then centrifuged (500 ×g for 10 min; 4°C) and the supernatant was frozen at -70°C. Slides were prepared using a Cytospin instrument and stained with May-Grunwald-Giemsa stain to permit differential cell counting. Slides with > 30% squamous cells were rejected. Initially, the absolute number of cells was counted. The percentage of neutrophils, eosinophils and lymphocytes was subsequently calculated to allow definition of the nasal mucosa inflammatory. Eosinophilic inflammation was considered to be present when the percentage of eosinophils was ≥ 1%.

### Skin prick tests

Because of its influence on exhaled nitric oxide levels, atopic status was recorded. Atopic status and sensitization to occupational allergens were assessed by skin prick tests (SPTs) performed on the forearm with a standard battery of common allergens and allergens found specifically in the working environment (Stallergenes Laboratories, Fresnes, France; Allerbio Laboratoire, Vandeuil, France). *Common allergens *included: (i) acarians (*Dermatophagoides pteronyssinus*, *D. farinae*), (ii) animal danders (cat, dog...); (iii) pollens (12 Graminaceae, mixed trees, mixed cereals, mixed grass, mixed betulaceae); and (iv) molds (*Alternaria tenius, Aspergillus fumigatus*). *Occupational allergens *included baking-related antigens (wheat flour, rye flour, oat flour, barley flour, α amylase and baker's yeast) and hairdressing-related antigens (1% solution of alkaline persulfates produced by the Center for Clinical Investigation pharmacy). A 10% histamine solution was used as a positive control and a phenolated 4‰ isotonic solution as a negative control. The largest heal diameter was assessed after 20 min. A positive SPT was defined as a wheal diameter equal to or greater than 3 mm with no reaction to negative control and positive reaction to histamine [10,35]. Atopy was defined as a positive response to at least one common allergen. New specific SPT reactivity was defined as a negative to positive change in skin reactivity.

### Statistical analyses

Statistical analysis was carried out using the SAS package. Personal characteristics such as gender, age, training track and smoking habits were compared at baseline between apprentices whose participation was complete along the study and subjects lost along follow-up. Medical characteristics such as atopy, respiratory and skin symptoms and AHR based on the last data collected before quitting the study were also compared between these two groups. Comparisons used chi-square or Student tests as appropriate.

The analysis that will be conducted to answer the main objective of this study rests on the following rationale. The "study outcome" is based on the metacholine challenge tests, as defined earlier. Then, the association between this outcome and each non-invasive test (including clinical symptoms) will be assessed, first at the last visit, then (among those associated at V4) at visits 3 and 2, in order to identify the one that will show most predictive (and soon occurring) of the final "study outcome", with consideration of possible heterogeneity according to atopy and apprentice track. Along the same line, optimal combinations of tests will also be explored.

## Results: participation and follow-up rates

Of 1839 apprentices invited to participate in the study from 6 apprenticeship schools, 1399 either refused or were excluded for not meeting the inclusion criteria. Enrolment of first graders was repeated for three successive years to reach the required sample size. A total of 441 subjects, representing 24.0% of those eligible, gave their consent and were included; 90 (20.0%) quit for several reasons that are given in the following section. Visit 2 was attended by 315 subjects (technical problems with the FE_NO _analyzer precluded invitation of students to this visit), V3 by 384 and the last visit by 354 students. Because of technical and funding problems at study inception, the second examination was not conducted for students recruited during the first year of the study, which explains the lower number of subject present at visit 2. Also, SPTs could not be done during the first visit during the first year of the study.

Great care was given to assessing reasons for follow-up loss. Among the 90 subjects who did not attend visit 4, 77 were interviewed by telephone; the remainder (14.4%) could not be found on telephone lists (change of civil name after marriage of girls, moving away from the study region). The main reasons invoked were departure from the training programme and lack of time (Table 1). Only 1 student said he had a respiratory condition. All characteristics we compared between participants and subjects lost along follow-up are similar (Table 2) except one: apprentices who were lost along follow-up tended to be more frequently smokers (57.8% versus 41.3% respectively, p = 0.005), but not heavier smokers (8.9 (± 5) cigarettes per day versus 9.9 (± 7.5) cigarettes per day respectively; p = 0.32).

**Table 1:** Reasons for loss along follow-up

**Reasons**	**N**	**Percent**
Lack of time	28	36.4
Medical examinations considered "painful"	1	1.3
Moving away from region	1	1.3
Other reasons	10	13.0
Quit training	37	48.1
*Reasons for leaving (among 37 quitters):*		
*dis interested in training*	*13*	*35.1*
*disagreement with manager*	*19*	*51.4*
*moving away*	*2*	*5.4*
*Pregnancy*	*1*	*2.7*
***respiratory or allergic conditions***	*1*	*2.7*
*other health problems (unrelated)*	*1*	*2.7*

**Total**	77	100

**Table 2:** Comparison of socio-demographic characteristics between lost subjects and the others participants

	**Subjects lost**	**Other participants**	**p**
Size, n (%)	90 (20.4%)	351 (79.6%)	
Age, mean (SD; in years)	17,0 (1.4)	16,9 (1.4)	0.33
Sex			0.16
Male, n (%)	57 (63.3%)	194 (55.3%)	
Female, n (%)	33 (36.7%)	157 (44.7%)	
Apprentices			0.16
Bakers, n (%)	39 (44.3%)	122 (34.8%)	
Pastry-makers, n (%)	24 (26.7%)	87 (24.8%)	
Hairdressers, n (%)	27 (30.0%)	142 (40.4%)	
Atopic status			0.27
Atopy, n (%)	45 (50.0%)	198 (56.4%)	
Non atopy, n (%)	45 (50.0%)	153 (43.6%)	
Active cigarette smoking			0.005
Smokers, n (%)	52 (57.8%)	145 (41.3%)	
Non-smokers, n (%)	38 (42.2%)	206 (58.7%)	

## Discussion

All the investigations planned in this study have been completed. However, the study lasted a year longer than expected because recruitment of volunteers was more difficult than anticipated. The discussion will focus on whether the final population sample is of sufficient quality to allow drawing valid conclusions.

The 24% participation rate can be considered fair. In a study among 346 bakery apprentices by Skjold et al, 54% of invited subject attended the first examination [36]. Our lower participation rate is not likely to be related to the nature of the medical examinations, which were quite similar in both studies, with the exception of FOT, only realized in our study, and specific IgE (thus blood sampling) tested only in Skjold's study. Instead, other factors could be invoked. For instance, one explanation might be the period of recruitment: while our study invited first graders, Skjold et al recruited students in the second year of training, when they know better their employer and can more easily request his/her agreement. In addition, in our study, some apprentices reported factors precluding inclusion such as asthma at the inclusion visit or previous exposure to agents known to induce OA. On the other hand, many apprentices chose not to enroll because of time constraints (each visit and the associated transport time took a whole school day), even though they were willing to take part. Finally, many apprentices and parents feared they would be asked to cease apprenticeship should sensitization to occupational allergens be diagnosed during the medical visits, although they were informed, both by flyers and during meetings, that this was not the purpose of the study. Such reluctance was also reported by Skjold et al, together with fear of blood tests and duration of follow-up (three years) [36]. Obviously, such factors are less likely to prevent workers to attend workplace health examinations, a setting in which our non-invasive investigations are designed to be used if their suitability is proven. Indeed, only one subject refused participation because of the nature of the proposed investigations, a fact underscoring their acceptability. Further, only the investigation (or a combination of tests) deemed most predictive of the final "study outcome" is liable to be proposed for future field screening procedures, meaning that the time required for its (their) implementation will be much shorter than needed for the present study.

Recruitment was the most difficult task. A "call center", which telephoned to students and parents if the consent form had not been returned, was set up when the study was already in progress. This considerably increased the number of signed consents (respectively 74, 156, 212 were returned for the three inclusion years). Although time-consuming and expensive – calls were given between 5 and 8:30 pm to enhance the probability of finding parents at home -, the "call center" was decisive in improving study recruitment. Because of the important number of medical examinations that were to be performed, it was not possible to include more than ten subjects a day thus lengthening the inclusion period up to 3 or 4 months. Further, starting with the second year of recruitment, both the visits of the first graders (V1) and V3 for the second graders (i.e. the cohort included the year before) had to be planned in sequence during fall and early winter, with the latter being done in priority. Thus, a whole cohort could have its first visit between December and March, with a few subjects being seeing as late as April. While this was not looked for in the study design, this feature presents a *post hoc *advantage in that it will allow to compare results of clinical and biological investigations among students who have been investigated « early » (operational definition: prior to January the 15^th^) and « late » (after January the 15^th^), i.e. respectively less than about 3 months and more than 3 months after inception of the training programme and associated exposures. This might permit to point out some differences in the dynamic process of sensitization between low molecular and high molecular weight agents, and cast hypotheses on the "allergic march".

Our observed drop-out rate was low (20%). The main explanation given by apprentices who did not show up at visit 4 was its close proximity with the final training tests, so they preferred not to waste this day during their exam preparation. One subject (a baker) abandoned the study because of skin allergy problems. This skin reaction occurred at the investigation centre well after termination of prick testing and just after lunch, suggesting it was probably not related to the allergens used in the study. Another subject quit because of infectious mononucleosis. Except for the proportion of smokers, no difference was observed in clinical and demographic characteristics between subjects lost for follow-up and those who remained in the study. We conclude that a selection bias along this prospective study is unlikely and that a healthy worker effect is more likely to play a role when deciding to enroll in the apprenticeship programme rather than by quitting it, at least among our volunteers [37,38]

The tests were generally well accepted. Nasal lavage – which was contra-indicated for subjects with latex allergy or nasal bleeding during the two days prior to the study – was found unpleasant by 3% of subjects, who declined its performance. Moreover, the test was not recommended for subjects currently using nasal drops or suffering from rhinitis with copious mucous secretion because this hampers cell counting. There were no formal contra-indications for other tests, which were subject to recommendations having on the whole no impact on their implementation. Combined with their capacity to correctly detect airways inflammation, good acceptability is of paramount importance if these tests are to be used to investigate early OA in field epidemiological studies or occupational health surveillance.

Individual exposure assessment for all apprentices was not feasible because of financial restrictions and mostly because they worked in shops and facilities scattered over a wide area. However, we investigated exposure levels among 62 apprentices during a whole work shift at two different seasons. The design and main results have been described separately for hairdressers [39] and for bakers and pastry makers [40]. Hairdressing apprentice exposures were lower than current threshold limit values for ammonia, hydrogen peroxide and persulfates. However, over half the technical areas, where dying, permanenting or bleaching chemical are handled, had no ventilation system [39]. Bakery and pastry apprentices experienced exposures to flour dust very close to the threshold limit value recommended by the ACGIH (American Conference of Governmental Industrial Hygienists) for inhalable flour dust in order to protect against sensitization and other respiratory symptoms [41], and this is of concern [40]. These findings may relate to observations made by several authors. Concerning respiratory condition prevalence or incidence, a recent study described respiratory symptoms (shortness of breath, wheezing, exercise induced symptoms) amongst pastry-maker apprentices [42]. Cough, dyspnoea, rhinitis, conjunctivitis and positive skin prick tests for flour allergens were reported among bakery apprentices [35,43-46]. These authors concluded that these tests should be performed with common and occupational allergens at the very start of apprenticeship, to identify subjects at risk of sensitization [35,47]. The occurrence of specific IgE antigens during the follow-up of bakery apprentices was described in another study [44]. Incidence of OA and occupational rhinitis has been shown to increase with exposure time [48]. There are few studies among hairdressing apprentices, but these young workers are exposed to the same substances as older personnel. Peak expiratory flow variability has been assessed in Italy for a 10-day period and has been reported with reference to job tasks [49]. A survey in Turkey measured a 1.7% prevalence of OA among 116 apprentices [50]. Hairdressing apprentices exhibited poorer lung function values than office apprentices in a French study [38]. These studies underline that early warnings of airways inflammation, suitable for field investigation, would be useful tools in preventing OA. The present study has been designed to contribute to assess such tools.

The risk of OA has been reported to manifest soon after first exposure, within 1 to 2 years [3]. The latency period, however, may span from months to years [51]. The rate of acquiring both sensitization and asthma-like symptoms might differ according to the nature of the agent [9] and the intensity of exposure [3]. While Malo et al. [52], and Nadeau et al. [53] showed that OA with latency period occurs during the first year of exposure to LMW agents in about 40% of cases, Skjold et al found peak values of sensitization to occupational HMW agents after four months of exposure among Danish bakery apprentices [44]. Our study will allow comparison of sensitization rates for both types of agents, which might shed additional light on the sequence of events leading from initial exposure to symptoms and eventually to disease.

In conclusion, this study has proved feasible. Recruitment has been completed. The data analysis is underway to identify among the set of non invasive investigations that were implemented the one (or the combination of tests) which will show most predictive of the airways inflammation at the last visit.

## Abbreviations

OA: occupational asthma; HMW: high molecular weight; LMW: low molecular weight; ONAP: observatoire national des asthmes professionnels; FOT: forced oscillation technique; FENO: fraction exhaled nitric oxide; AHR: airways hyper responsiveness; RADS: reactive airways dysfunction syndrome; ROS: Random-noise Oscillatory Spirometer; MCT (+): methacholine challenge test (positive); FVC: forced vital capacity; FEV1: forced expiratory volume in 1 second; V'max: maximal expiratory flows at various lung volumes; DRS: dose-response slope; SPTs: skin prick tests; ACGIH: American Conference of Governmental Industrial Hygienists; AFSSET: Agence Française de Sécurité Sanitaire de l'Environnement et du Travail; CRAM: Caisse Régionale d'Assurance Maladie; INRS: Institut National de Recherche et de Sécurité pour la prévention des maladies professionnelles et des accidents du travail; INSERM: Institut National de la Santé et de la Recherche Médicale.

## Competing interests

The authors declare that they have no competing interests.

## Authors' contributions

PT substantial contribution to data acquisition, analysis and interpretation; involved in writing the article. AB study design, data collection, analysis and interpretation; involved in writing the article. VD data acquisition; critically reviewing the draft for important intellectual content. PW study design, critically reviewing the draft for important intellectual content. J-PM data acquisition, analysis and interpretation. BH data acquisition; critically reviewing the draft for important intellectual content. CP data acquisition; critically reviewing the draft for important intellectual content. DZ-N study design, design of statistical analysis and interpretation; involved in writing the article.

## Pre-publication history

The pre-publication history for this paper can be accessed here:


